# Dias MBK, Assis M, Santos ROM, Ribeiro CM, Migowski A, Tomazelli JG. Adequação da oferta de procedimentos para a detecção precoce do câncer de mama no Sistema Único de Saúde: um estudo transversal, Brasil e regiões, 2019. Cad Saúde Pública 2024; 40(5):e00139723.

**DOI:** 10.1590/0102-311XER139723

**Published:** 2024-07-29

Onde se lê:

*Abstract*

Early detection is a major strategy in breast cancer control and, for this reason, it is important to ensure access to investigation of suspected cases for care continuity and timely treatment. This study aimed to estimate the need for procedures of breast cancer early detection and assess their adequacy for providing care to screened and symptomatic women in the Brazilian Unified National Health System (SUS) in 2019. A descriptive cross-sectional study was conducted to analyze the provision of tests for breast cancer early detection, comparing the estimated need with the procedures performed in the SUS. Parameters provided by the Brazilian National Cancer Institute were used to estimate the population and the need for early detection tests. The number of procedures performed in 2019 was obtained from the Outpatient Information System of the SUS. A deficit in screening mammograms was observed in the country (-45.1%), ranging from -31.4% in the South Region to -70.5 % in the North Region. If this test was offered to the target population, the deficit in the country would reduce to -14.8% and there would be an oversupply in the South Region (6.2%). Diagnostic investigation procedures varied between the regions, with higher deficits in coarse needle biopsy (-90.8%) and breast lump biopsy/excision (-80.6%) observed in the Central-West Region, and the highest deficit in anatomopathological exams in the North Region (-88.5%). The comparison between the production and need for procedures of breast cancer early detection in Brazil and its regions identified deficits and inadequacies that must be better understood and addressed at the state and municipal levels.

Leia-se:

*Abstract*

Early detection is a major strategy in breast cancer control and, for this reason, it is important to ensure access to investigation of suspected cases for care continuity and timely treatment. This study aimed to estimate the need for procedures of breast cancer early detection and assess their adequacy for providing care to screened and symptomatic women in the Brazilian Unified National Health System (SUS) in 2019. A descriptive cross-sectional study was conducted to analyze the provision of tests for breast cancer early detection, comparing the estimated need with the procedures performed in the SUS. Parameters provided by the Brazilian National Cancer Institute were used to estimate the population and the need for early detection tests. The number of procedures performed in 2019 was obtained from the Outpatient Information System of the SUS. A deficit in screening mammograms was observed in the country (-45.1%), ranging from -31.4% in the South Region to -70.5 % in the North Region. If this test was offered to the target population, the deficit in the country would reduce to -14.8% and there would be an oversupply in the South Region (6.2%). Diagnostic investigation procedures varied between the regions, with higher deficits in core needle biopsy (-90.8%) and breast lump biopsy/excision (-80.6%) observed in the Central-West Region, and the highest deficit in anatomopathological exams in the North Region (-88.5%). The comparison between the production and need for procedures of breast cancer early detection in Brazil and its regions identified deficits and inadequacies that must be better understood and addressed at the state and municipal levels.

Onde se lê:

*Resumen*

La detección temprana es una de las estrategias para el control del cáncer de mama y, para ello, es fundamental garantizar el acceso a la investigación de los casos sospechosos para la continuidad del cuidado y el tratamiento oportuno. El presente estudio tiene como objetivo estimar la necesidad de procedimientos para la detección temprana de esta neoplasia y evaluar su adecuación en la atención a las mujeres rastreadas y sintomáticas en el Sistema Único de Salud (SUS) brasileño, en el año 2019. Se realizó un estudio descriptivo transversal para analizar la oferta de pruebas para la detección temprana del cáncer de mama, comparando la necesidad estimada con los procedimientos realizados en el SUS. Se utilizaron los parámetros proporcionados por el Instituto Nacional del Cáncer para estimar la población y la necesidad de pruebas para la detección temprana. El número de procedimientos realizados en el 2019 se obtuvo del Sistema de Información Ambulatoria del SUS. Se observó un déficit de mamografías de tamizaje en el país (-45,1%), oscilando entre el -31,4% en la Región Sur y el -70,5% en la Región Norte. Si la oferta de esta prueba se dirigiera a la población objetivo del rastreo, el déficit en el país se reduciría al -14,8% y habría una sobreoferta en el Sur (6,2%). Los procedimientos de investigación diagnóstica presentaron variaciones entre regiones, observándose mayores déficits en punción con aguja gruesa (-90,8%) y biopsia/escisión de nódulo mamario (-80,6%) en el Centro-Oeste, y el mayor déficit de pruebas anatomopatológicas en el Norte (-88,5%). La comparación entre la producción y la necesidad de procedimientos para la detección temprana del cáncer de mama en Brasil y en las regiones identificó déficits e insuficiencias que deben ser mejor conocidos y abordados a nivel estatal y municipal.

Leia-se:

*Resumen*

La detección temprana es una de las estrategias para el control del cáncer de mama y, para ello, es fundamental garantizar el acceso a la investigación de los casos sospechosos para la continuidad del cuidado y el tratamiento oportuno. El presente estudio tiene como objetivo estimar la necesidad de procedimientos para la detección temprana de esta neoplasia y evaluar su adecuación en la atención a las mujeres rastreadas y sintomáticas en el Sistema Único de Salud (SUS) brasileño, en el año 2019. Se realizó un estudio descriptivo transversal para analizar la oferta de exámenes para la detección temprana del cáncer de mama, comparando la necesidad estimada con los procedimientos realizados en el SUS. Se utilizaron los parámetros proporcionados por el Instituto Nacional del Cáncer para estimar la población y la necesidad de exámenes para la detección temprana. El número de procedimientos realizados en el 2019 se obtuvo del Sistema de Información Ambulatoria del SUS. Se observó un déficit de mamografías de tamizaje en el país (-45,1%), oscilando entre el -31,4% en la Región Sur y el -70,5% en la Región Norte. Si la oferta de este examen se dirigiera a la población objetivo del rastreo, el déficit en el país se reduciría al -14,8% y habría una sobreoferta en el Sur (6,2%). Los procedimientos de investigación diagnóstica presentaron variaciones entre regiones, observándose mayores déficits en punción con aguja gruesa (-90,8%) y biopsia/escisión de nódulo mamario (-80,6%) en el Centro-Oeste, y el mayor déficit de exámenes anatomopatológicas en el Norte (-88,5%). La comparación entre la producción y la necesidad de procedimientos para la detección temprana del cáncer de mama en Brasil y en las regiones identificó déficits e insuficiencias que deben ser mejor conocidos y abordados a nivel estatal y municipal.

